# Elasmobranch bycatch in the demersal prawn trawl fishery in the Gulf of Papua, Papua New Guinea

**DOI:** 10.1038/s41598-019-45715-w

**Published:** 2019-06-25

**Authors:** W. T. White, L. Baje, C. A. Simpfendorfer, S. A. Appleyard, A. Chin, B. Sabub, E. Rochel, G. J. P. Naylor

**Affiliations:** 1 CSIRO Australian National Fish Collection, National Research Collections Australia, Castray Esplanade, Hobart, 7001 Tasmania Australia; 2 CSIRO Oceans & Atmosphere, Castray Esplanade, Hobart, 7001 Tasmania Australia; 3 Papua New Guinea National Fisheries Authority, P.O. Box 2016, Port Moresby, National Capital District Papua, New Guinea, Australia; 4 Centre for Sustainable Tropical Fisheries and Aquaculture & College of Science and Engineering, James Cook University, Townsville, Queensland 4108 Australia; 5 College of Charleston, Hollings Marine Laboratory, 331 Fort Johnson Road, 29412 Charleston, SC USA; 6 Florida Museum of Natural History, University of Florida, Gainesville, FL 32611 USA

**Keywords:** Ecology, Zoology

## Abstract

The elasmobranch bycatch of the Gulf of Papua Prawn Fishery is investigated in detail for the first time. Fisheries observers collected data on the elasmobranch bycatch from a total of 403 trawl sets (1,273 hrs) in the Gulf of Papua. A total of 40 species of elasmobranchs were recorded ranging in size from a 12 cm disc width stingray to a 350 cm total length sawfish. High mortality rates were recorded (>80%), attributed to the long trawl durations (up to 4 hours). The future inclusion of bycatch reduction devices would likely reduce the number of larger elasmobranchs being caught, based on evidence from the prawn trawl fisheries of northern Australia, and is being investigated by the PNG National Fisheries Authority. Differences in catch compositions were detected across the management zones as well as between the two monsoonal seasons (SE Monsoon and NW Monsoon). Increased monitoring and additional research is required and management plans should address the elasmobranch bycatch and in particular their high mortality rate.

## Introduction

The majority of the global elasmobranch catch is incidental, in the form of bycatch from fisheries that target teleosts or crustaceans^1,2^. Accurate catch composition data are often not available and, particularly in developing countries, there are few management regulations or reporting requirements for elasmobranchs, and even less so for bycatch^3^. Large declines in abundance of demersal elasmobranchs have been recorded from several locations in the Indo–West Pacific, e.g. Thailand^4,5^ and Indonesia^6^. For example, in the Andaman Sea region of Thailand, a comprehensive survey of sharks observed at fish landing sites in 2014 and 2015 recorded far less landings of larger sharks compared to a 2004 survey^5^. Similarly, a study on elasmobranch fisheries in southern Indonesia highlighted that catches of elasmobranchs in the Java Sea had declined by at least one order of magnitude between 1976 and 1997^6^. However, there has been very few studies on the elasmobranch bycatch in most demersal fisheries in the tropical Indo–West Pacific. Elasmobranchs are important apex predators in marine ecosystems, but many populations are under significant pressure from overexploitation^4^. Elasmobranchs typically have a *k*-selected life history, i.e. slow growth rates, low fecundity, late maturity and long gestation period, which leads to them having low productivity^4^. Thus, it is important to obtain information on catch composition of elasmobranch bycatch from fisheries, to provide the evidence-based science required for fisheries assessments.

Approximately 44% of annual global tropical prawn catches come from the Coral Triangle region, with the majority coming from Indonesia (18% of global catch)^7^. Although Papua New Guinea (PNG) contributes only about 0.1% to the global tropical prawn catches, it is the only Pacific Island nation with a demersal prawn trawl fishery. Despite its small size, it is one of the most valuable export fisheries for PNG, earning revenue of ~K10 million (~$3 USD) annually^8^. No foreign trawl vessels currently operate in PNG waters.

The first surveys for prawns in the Gulf of Papua (GoP) were carried out in 1954, while commercial fishing explicitly targeting prawn commenced in 1969^9,10^. The GoP prawn fishery currently extends along the south coast of PNG from the mouth of the Fly River in the west to the Iokea coast in the east to depths of about 40 m^8^. The main prawn species targeted are banana prawns *Penaeus merguiensis* and Indian white prawn *P. indicus*, with lower catches of giant tiger prawns *P. monodon* and green tiger prawn *P. semisulcatus*^8^. Although an estimated 9,603 km^2^ of the GoP is considered suitable for trawling, 1,388 km^2^ receives more than 50% of the total fishing effort (hours of trawling)^8^. The majority of fishing effort is in Kerema Bay and Orokolo Bay which are around a 20 hr steam from Port Moresby. The GoP prawn fishery currently is limited to 15 licenses, although only 6 vessels operated in 2014 and 2015^8^ (NFA unpubl. data). Between 1990 and 2011, the average annual catch of prawns in the GoP prawn fishery was 625.9 mt^8^.

Although there are detailed data on the teleost bycatch in the GoP prawn fishery^11^, these data included no information on the elasmobranch bycatch. Prior to the current study, no data existed on the elasmobranch bycatch of this fishery both in terms of catch composition but also the fate of the bycatch following capture. Between 2014 and 2015, PNG’s National Fisheries Authority (PNG NFA) ran an observer program to investigate the elasmobranch bycatch in the GoP prawn fishery. The current study provides the first detailed investigation of this bycatch in PNG, including species, sex and size composition, and address the question of whether species richness and abundance varies at a spatial and temporal level. The major aim of this study was to provide management options to PNG’s NFA relating to the elasmobranch bycatch of GoP prawn fishery based on evidence-based science.

## Results

### Sampling effort

**Figure 1:**
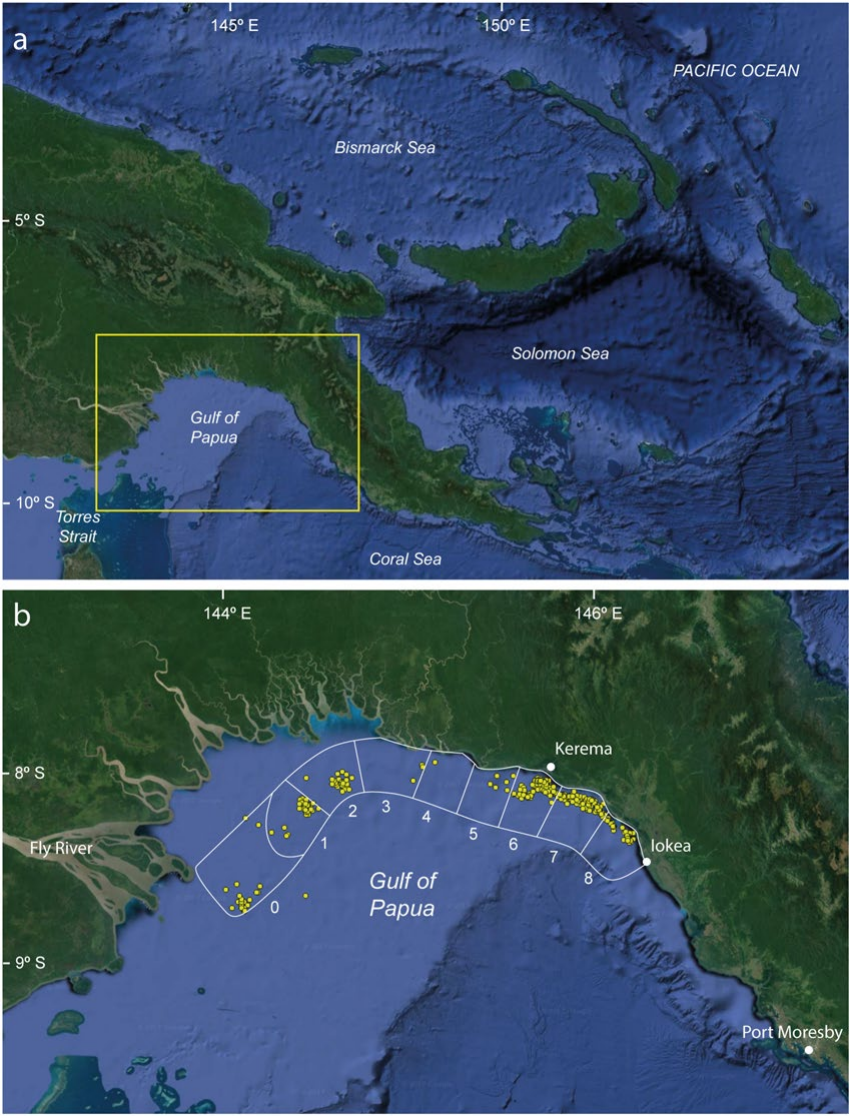
Map of Papua New Guinea: (**a**) whole country with yellow box indicating the Gulf of Papua region; (**b**) Gulf of Papua showing the locations of the trawl sets observed in this study superimposed over the fisheries management zones (1–8) and the extralimital Fly zone (0). Map data: ©2017 Google Earth, NASA; ©2013, TerraMetrics, Inc. www.terrametrics.com.

A total of 7 fishing trips were observed in this study. Five of the fishing trips comprised 9–17 days of trawling activity, and two comprised 35 and 36 days of trawling. The latter two longer fishing trips were not planned to be long trips prior to the departure from Port Moresby in September 2015 but both skippers remained fishing for a long period. It was not determined why these trips were longer than the other fishing trips observed. Data were obtained based on catches from 1,273 hours of trawling in 403 trawls within the GoP carried out between June 2014 and August 2015 at depths of 6 to 37 m. Although trawls were carried out across all fishery management zones in the GoP (Fig. 1), effort was not evenly distributed across them (Table 1). Trawling was concentrated in fisheries management zone 6 (n = 146 trawls), followed by zones 7 (n = 97 trawls) and 2 (n = 67 trawls), equating to 77% of all trawls being recorded from these three zones. Trawling in each zone covered roughly similar depth ranges. The majority of observed trawls were carried out during the day (6:00 am to 5:59 pm, n = 282) compared to at night (6:00 pm to 5:59 am, n = 120), i.e. 282 vs 120 respectively. The majority of trawls (64%, n = 258) were conducted during the Southeast Monsoon (SE), i.e. between May and September. This difference reflects the closure of fishing zones 2–8 to trawling between 1^st^ December and 31^st^ March^12^ (during the Northwest Monsoon, NE).

**Table 1:** Number of trawls, number of hours trawled, depth range fished, number of elasmobranchs recorded and catch per unit effort (CPUE, number of elasmobranchs/hour of trawling) in each of the fishing zones in the Gulf of Papua.

Fishing zone	# trawls	# hours	depth range	# elasmos	CPUE (elasmos hr^−1^) ± SD
0	25	65.4	8–31	163	2.5 ± 1.9
1	6	11.2	7–36	28	2.5 ± 2.5
2	67	203.3	10–37	351	1.7 ± 1.3
3	1	3.0	16–18	10	3.3
4	3	9.5	11–34	0	0.0
5	17	40.0	6–24	51	1.3 ± 1.7
6	146	498.9	9–29	878	1.8 ± 3.0
7	97	320.9	8–26	314	1.0 ± 1.3
8	40	120.9	7–34	235	1.9 ± 1.4
Total	402	1273.1	6–37	2030	1.7 ± 2.2

A total of 2,030 elasmobranch specimens were recorded from 339 of the 402 (i.e. 84%) trawls observed. No elasmobranchs were recorded on the remaining 63 trawls observed. Most elasmobranchs were recorded in fishing zone 6 (n = 878), followed by zones 2 (n = 351) and 7 (n = 314). Overall catch per unit effort (CPUE), calculated as number of elasmobranchs caught per hour of trawling (in all trawls, i.e. including those with zero elasmobranchs), was 1.7 elasmobranch hr^−1^. There was a significant difference in CPUE between the zones (GLMM, *P* < 0.005), but not between season and time of day (*P* > 0.05). Post-hoc two-tailed t-tests with Holm-Bonferroni correction found that all but four two-tailed tests were not significant (*P* > 0.05), with only zone 7 vs. zones 0, 1, 2 and 8 differing significantly (*P* < 0.005). Post-hoc t-test also indicated that the western (zones 0–4) and eastern (zones 5–8) zones differed significantly (*P* < 0.005). CPUE was greatest in fishing zones 0 and 1 (2.5 elasmobranchs hr^−1^), and lowest in fishing zone 7 (1.0 elasmobranchs hr^−1^), with the exception of zone 4 where no elasmobranchs were caught during the three trawls observed (Table 1). The low CPUE in fishing zone 7 is the most likely cause of the significant pairwise differences found.

### Elasmobranch species diversity and biomass

A total of 40 species (18 sharks and 22 rays) and 14 families (5 shark and 9 ray) of elasmobranchs were recorded during the survey period (Table 2). An additional family (Ginglymostomatidae) and two additional species (*Nebrius ferrugineus* and *Urogymnus asperrimus*) were also verified from images from the bycatch of the GoP prawn fishery provided by other sources, but not during the observer trips in this study. These two species are included in Table 2, but are not included in any analyses or summaries below.

**Table 2:** Abundance (number), biomass (in kg), CPUE (elasmobranchs per 100 hrs), size range (TL, total length; DW, disc width) recorded and maximum known size of each elasmobranch species recorded in this study. The two species with an asterisk were recorded from this fishery outside of this study and were not used in the analyses.

Species	#	# (%)	Weight (kg)	Weight (%)	CPUE (#/100 hrs)	Size range (cm)	Max. known size (cm)
**Hemiscylliidae**							
*Chiloscyllium punctatum*	74	3.6	34.4	0.8	5.8	TL: 18–88	TL: 132
**Stegostomatidae**							
*Stegostoma fasciatum*	10	0.5	21.0	0.5	0.8	TL: 39–186	TL: 235
*Ginglymostomatidae							
*Nebrius ferrugineus	—	—	—	—	—	—	
**Hemigaleidae**							
*Hemigaleus australiensis*	118	5.8	40.0	0.9	9.3	TL: 21–90	TL: 110
**Carcharhinidae**							
*Carcharhinus amblyrhynchoides*	1	<0.1	4.5	0.1	0.1	TL: 87	TL: 178
*Carcharhinus amboinensis*	3	0.1	15.2	0.3	0.2	TL: 89–95	TL: 280
*Carcharhinus brevipinna*	20	1.0	88.8	2.0	1.6	TL: 79–158	TL: 300
*Carcharhinus coatesi*	192	9.5	201.9	4.6	15.1	TL: 33–88	TL: 88
*Carcharhinus fitzroyensis*	18	0.9	94.5	2.2	1.4	TL: 66–123	TL: 135
*Carcharhinus leucas*	3	0.1	66.1	1.5	0.2	TL: 85–192	TL: 340
*Carcharhinus limbatus*	11	0.5	20.1	0.5	0.9	TL: 55–91	TL: 250
*Carcharhinus macloti*	19	0.9	35.2	0.8	1.5	TL: 38–90	TL: 110
*Carcharhinus sorrah*	3	0.1	15.6	0.4	0.2	TL: 96–100	TL: 160
*Carcharhinus tilstoni*	8	0.4	33.8	0.8	0.6	TL: 54–139	TL: 200
*Rhizoprionodon acutus*	148	7.3	117.3	2.7	11.6	TL: 31–86	TL: 100
*Rhizoprionodon taylori*	597	29.4	356.6	8.2	46.9	TL: 30–68	TL: 68
**Sphyrnidae**							
*Eusphyra blochii*	86	4.2	164.7	3.8	6.8	TL: 37–159	TL: 186
*Sphyrna lewini*	133	6.6	162.6	3.7	10.4	TL: 40–171	TL: 350
*Sphyrna mokarran*	2	0.1	20.3	0.5	0.2	TL: 119–150	TL: 600
**Pristidae**							
*Anoxypristis cuspidata*	11	0.5	190.3	4.4	0.9	TL: 102–215	TL: 350
*Pristis pristis*	1	<0.1	126.4	2.9	0.1	TL: 349	TL: 656
**Rhinidae**							
*Rhina ancylostoma*	2	0.1	52.8	1.2	0.2	TL: 120–165	TL: 270
*Rhynchobatus palpebratus*	60	3.0	401.9	9.2	4.7	TL: 43–234	TL: 262
**Glaucostegidae**							
*Glaucostegus typus*	5	0.2	96.5	2.2	0.4	TL: 39–240	TL: 284
**Gymnuridae**							
*Gymnura australis*	154	7.6	149.8	3.4	12.1	DW: 26–77	DW: 94
**Dasyatidae**							
*Hemitrygon longicauda*	25	1.2	9.4	0.2	2.0	DW: 12–31	DW: 31
*Himantura australis*	13	0.6	402.7	9.2	1.0	DW: 52–140	DW: 183
*Himantura leoparda*	19	0.9	180.9	4.1	1.5	DW: 38–104	DW: 140
*Maculabatis astra*	134	6.6	293.7	6.7	10.5	DW: 22–76	DW: 92
*Megatrygon microps*	1	<0.1	80.0	1.8	0.2	DW: ~180	DW: 222
*Neotrygon annotata*	35	1.7	15.3	0.4	2.7	DW: 12–30	DW: 30
*Neotrygon picta*	1	<0.1	0.1	<0.1	0.1	DW: 14	DW: 32
*Pastinachus ater*	3	0.1	50.6	1.2	0.2	DW: 80–100	DW: 200
*Pateobatis fai*	3	0.1	140.0	3.2	0.2	DW: 67–170	DW: 170
*Pateobatis hortlei*	32	1.6	79.6	1.8	2.5	DW: 16–112	DW: 112
*Urogymnus acanthobothrium*	3	0.1	84.0	1.9	0.2	DW: 100–114	DW: 161
*Urogymnus granulatus	—	—	—	—	—	—	
unknown stingray	1	<0.1	12.0	0.3	0.1	DW: 78	—
**Myliobatidae**							
*Aetomylaeus caeruleofasciatus*	46	2.3	25.1	0.6	3.6	DW: 20–52	DW: 59
**Aetobatidae**							
*Aetobatus ocellatus*	5	0.2	45.1	1.0	0.4	DW: 66–107	DW: 300
**Rhinopteridae**							
*Rhinoptera neglecta*	29	1.4	293.2	6.7	2.3	DW: 37–140	DW: 140
**Mobulidae**							
*Mobula alfredi*	1	<0.1	145.8	3.3	0.1	DW: 220	DW: 550

The *a* and *b* parameters used to convert the estimated total lengths or disc widths (cm) to total weight (g) are provided in Table 3 together with their source. Although sharks represented 71% of the total elasmobranch abundance in the catches, they represented only 34% of the total biomass. The most species rich families were Carcharhinidae (whaler sharks) and Dasyatidae (stingrays) with 12 and 11 species, respectively.

**Table 3:** Length to weight conversion parameters, and their source, used to estimate biomass of specimens measured but not weighed.

Species	*a*	*b*	# based on	*R* ^2^	Source
*Chiloscyllium punctatum*	0.0043	2.9847	9	0.994	this study
*Stegostoma fasciatum*	0.0089	2.7313	4	0.997	this study
*Hemigaleus australiensis*	0.00348	3	425	0.982	^24^
*Carcharhinus amblyrhynchoides*	0.00265	3.21	67	0.975	^24^
*Carcharhinus amboinensis*	0.00194	3.27	104	0.986	^24^
*Carcharhinus brevipinna*	0.00317	3.1	507	0.874	^38^
*Carcharhinus coatesi*	0.0066	2.9286	127	0.979	this study
*Carcharhinus fitzroyensis*	0.00142	3.292	109	0.960	^39^
*Carcharhinus leucas*	0.0111	2.923	182	0.908	www.fishbase.org (for Mexico)
*Carcharhinus limbatus*	0.00251	3.125	183	0.989	^40^
*Carcharhinus macloti*	0.000391	3.55	127	0.830	^24^
*Carcharhinus sorrah*	0.000545	3.51	164	0.900	^41^
*Carcharhinus tilstoni*	0.00475	3.06	311	0.910	^41^
*Rhizoprionodon acutus*	0.0055	2.9298	64	0.989	this study
*Rhizoprionodon taylori*	0.0026	3.1558	185	0.989	this study
*Eusphyra blochii*	0.006	2.8748	50	0.974	this study
*Sphyrna lewini*	0.00399	3.03	252	0.985	^42^
*Sphyrna mokarran*	0.00123	3.24	117	0.991	^42^
*Anoxypristis cuspidata*	0.05	2.4735	45	0.855	^43^
*Pristis pristis*	0.003	2.9985	23	0.949	^43^
*Rhina ancylostoma*	0.008	3.012	6	0.999	from length and weights in^44–47^.
*Rhynchobatus palpebratus*	0.0045	2.9959	21	0.987	this study
*Glaucostegus typus*	0.0046	2.9184	309	0.980	W. White (unpubl. data)
*Gymnura australis*	0.0055	3.108	49	0.961	this study
*Hemitrygon longicauda*	0.1708	2.5027	4	0.890	this study
*Himantura australis/leoparda*	0.0728	2.7578	6	0.974	this study
*Maculabatis astra*	0.0219	3.0471	46	0.984	this study
*Neotrygon annotata*	0.0437	2.8717	26	0.802	this study
*Pateobatis hortlei*	0.0765	2.174	14	0.928	this study
*Aetomylaeus caeruleofasciatus*	0.007	3.1539	9	0.977	this study
*Aetobatus ocellatus*	0.0276	2.87	331	0.980	^48^(for *A. narinari*)
*Rhinoptera neglecta*	0.0487	2.6886	5	0.998	this study

Six species represented 65% of the total elasmobranch catch (by abundance): *Rhizoprionodon taylori* (29.4%), *Carcharhinus coatesi* (9.5%), *Gymnura australis* (7.6%), *Sphyrna lewini* (6.6%), *Maculabatis astra* (6.6%) and *Hemigaleus australiaensis* (5.8%). Of the remaining species, 24 were rarely caught (<1% of total abundance). Five species represented 40% of the total elasmobranch biomass: *Rhynchobatus palpebratus* (9.2%), *Himantura australis* (9.2%), *Rhizoprionodon taylori* (8.2%), *Maculabatis astra* (6.7%) and *Rhinoptera neglecta* (6.7%). Of the remaining species, 14 represented <1% of total biomass.

A total of 81% of elasmobranchs recorded by observers were recorded as dead at capture, 15% as dying or moribund, and only ~4% as alive. The long average trawl duration is likely to the main contributor to the high mortality rate. The sharks and shark-like rays in the bycatch typically have their fins removed. Although large portions of the fish bycatch is discarded (mostly dead), in the more inshore areas close to major towns, such as Kerema, there is an arrangement with local villagers to access the bycatch. In this scenario, up to 10 small boats can pull up to the trawler with as many as 30–40 people coming on-board to divide up the bycatch from the trawl haul. Prior to this arrangement, inshore fishing by the trawlers was often met with hostility by the local villagers.

### Size compositions

The bycatch of sharks and rays in the GoP prawn trawl fishery encompassed a wide size range of individuals. Captured elasmobranchs ranged from newly hatched *Chiloscyllium punctatum* (18 cm TL) and presumably newborn *Hemitrygon longicauda* (12 cm DW), up to a 350 cm TL *Pristis pristis*. Thus, the catch composition from this fishery likely provides a reasonably comprehensive inventory of the sharks and rays occurring in the GoP.

**Figure 2:**
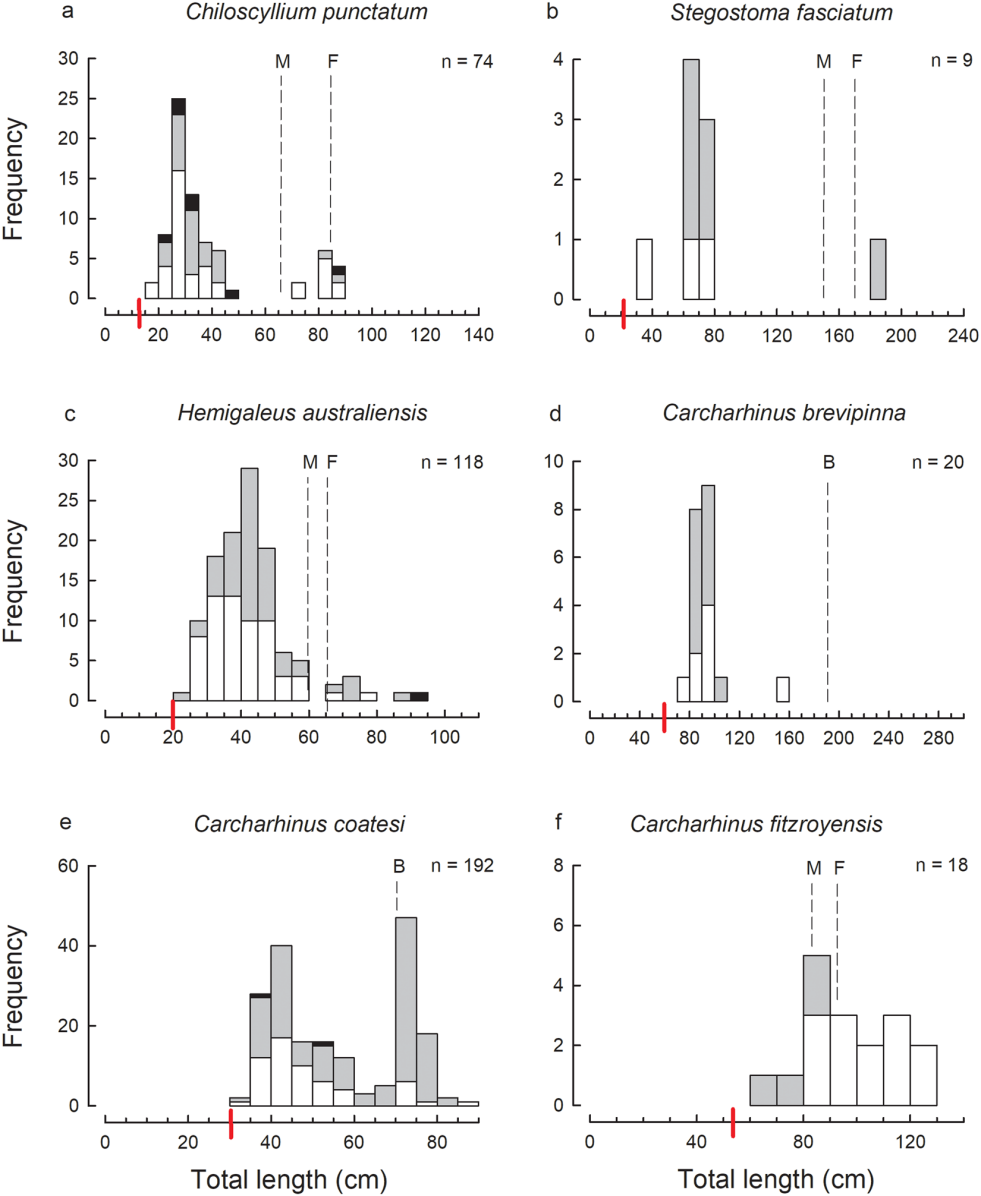
Size-frequency histograms of the most abundant shark species represented by 9 or more individuals in the trawl catches of the Gulf of Papua: (**a**) *Chiloscyllium punctatum*; (**b**) *Stegostoma fasciatum*; (**c**) *Hemigaleus australiensis*; (**d**) *Carcharhinus brevipinna*; (**e**) *Carcharhinus coatesi*; (**f**) *Carcharhinus fitzroyensis*. In this Figure and Figs 2–4, the species are placed in phylogenetic order from bamboosharks through to cownose rays; white bars denote females, grey bars males and black bars unsexed individuals; the total number (n) of individuals, known size at birth (red line) and known size at maturity (left dashed line denotes known size of maturity for males, right dashed line denotes known size at maturity for females; a single dashed line indicates both sexes mature at that size or known for only males in which case denoted with a ‘M’ above line) is given for each species; the size scale bar (x-axis) extends to the maximum known size for each of the species.

**Figure 3:**
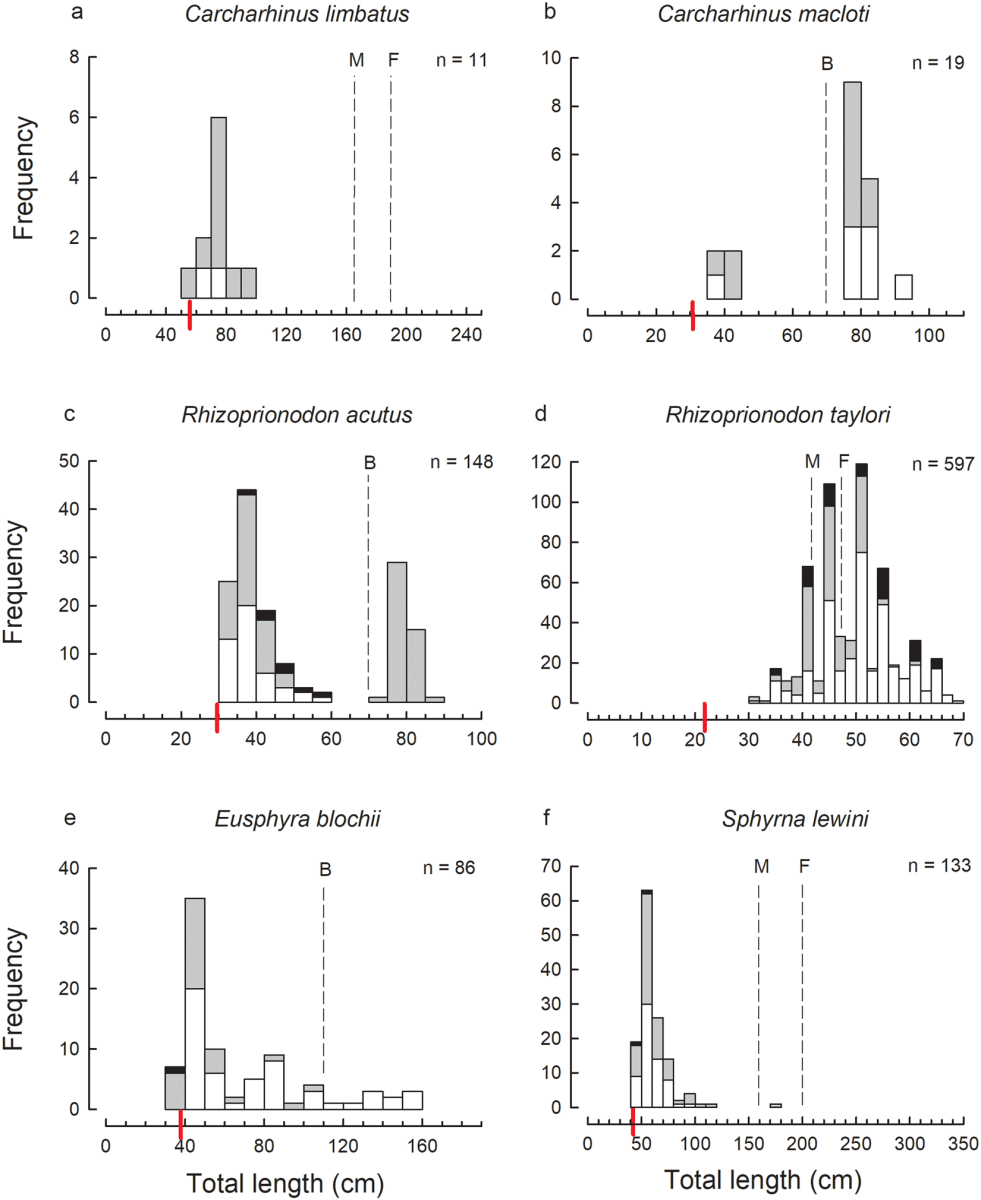
Size-frequency histograms of the most abundant shark species represented by 9 or more individuals in the trawl catches of the Gulf of Papua: (**a**) *Carcharhinus limbatus*; (**b**) *Carcharhinus macloti*; (**c**) *Rhizoprionodon acutus*; (**d**) *Rhizoprionodon taylori*; (**e**) *Eusphyra blochii*; (**f**) *Sphyrna lewini*.

**Figure 4:**
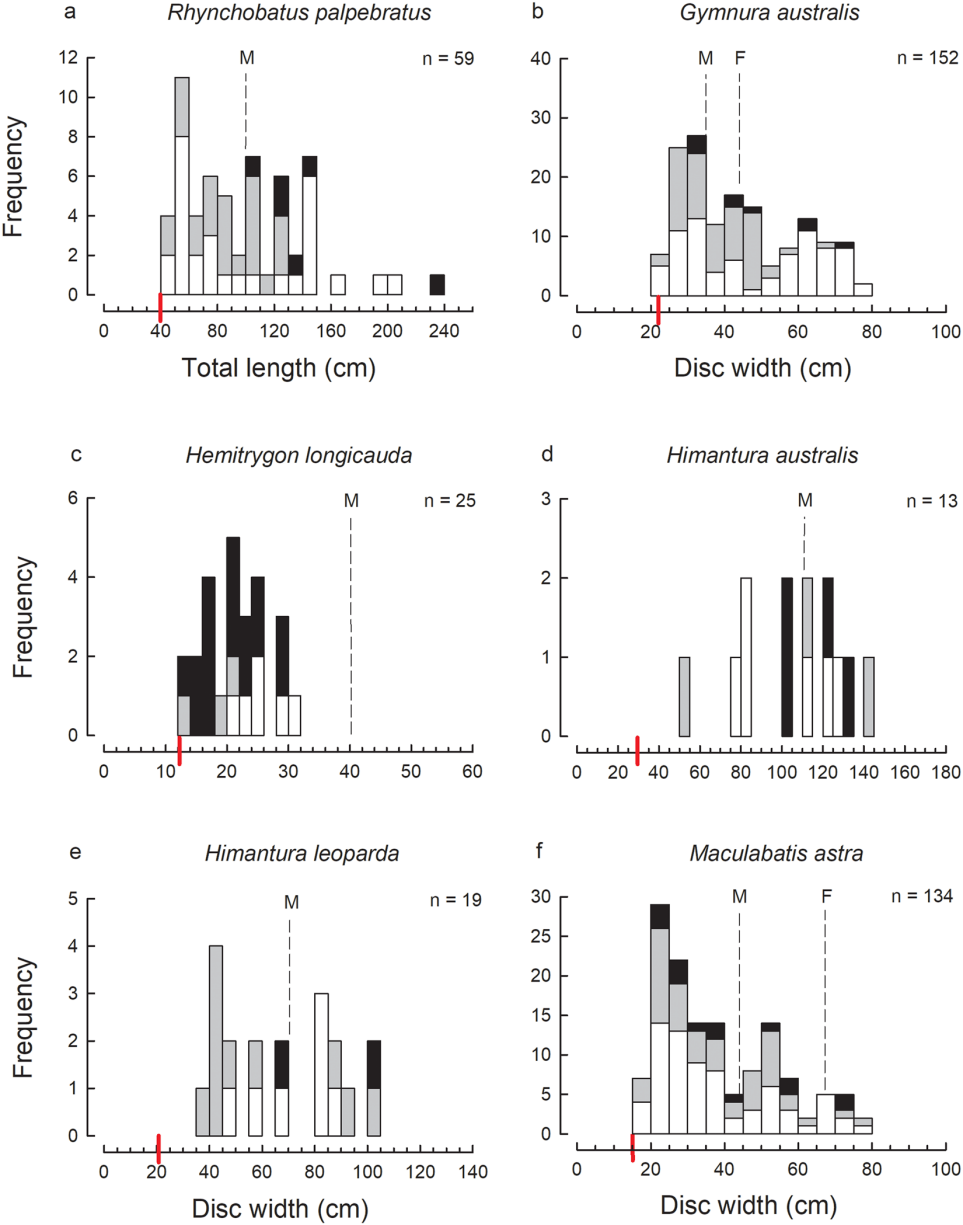
Size-frequency histograms of the most abundant ray species represented by 9 or more individuals in the trawl catches of the Gulf of Papua: (**a**) *Rhynchobatus palpebratus*; (**b**) *Gymnura australis*; (**c**) *Hemitrygon longicauda*; (**d**) *Himantura australis*; (**e**) *Himantura leoparda*; (**f**) *Maculabatis astra*.

**Figure 5:**
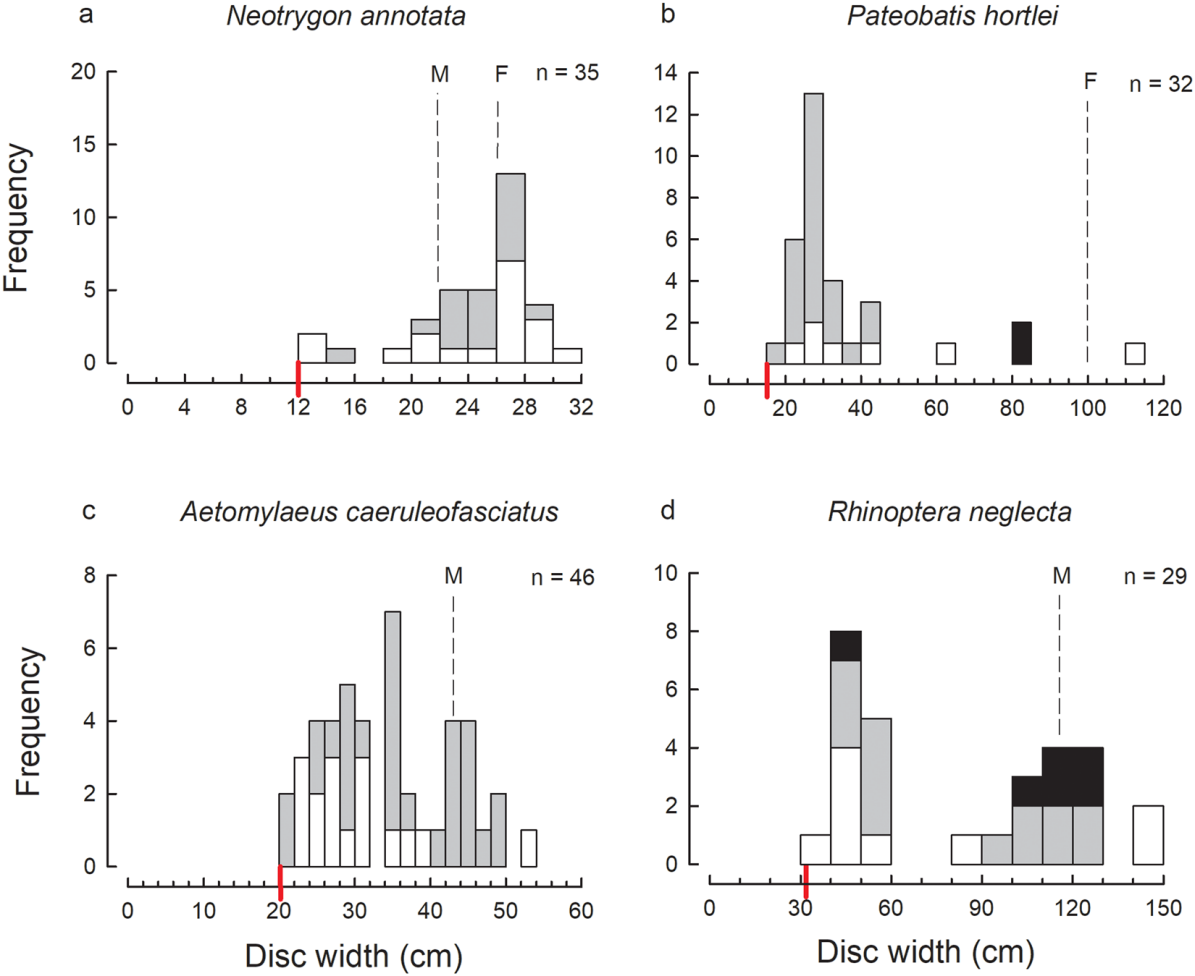
Size-frequency histograms of the most abundant ray species represented by 9 or more individuals in the trawl catches of the Gulf of Papua: (**a**) *Neotrygon annotata*; (**b**) *Pateobatis hortlei*; (**c**) *Aetomylaeus caeruleofasciatus*; (**d**) *Rhinoptera neglecta*.

Based on the size-frequency data for the most abundant species (Figs 2–5), bycatch from the trawl fishery included juveniles close to birth size for all species, except *R. taylori*. The latter species has a birth size of 22–26 cm TL^13^ but the smallest specimens recorded were in the 30–32 cm length class. The newborns of this species are very slender and may escape capture by trawl nets, or alternatively may occur in different areas or position in the water column thus evading capture. The capture of late-term pregnant females indicates that they give birth in the GoP and so the newborn individuals are expected to occur within the trawled area.

For several species, only immature individuals were recorded, e.g. *Carcharhinus brevipinna*, *C. limbatus*, *Sphyrna lewini*, *Hemitrygon longicauda* (Figs 2–4). Note that in this case and below, determination of immature individuals for each species is based on the theoretical size-at-maturity (see Figs 2–5) and not sampling observations from this study as not all individuals were examined for maturity. The catches of several of the remaining abundant species consisted of mostly immature individuals with only a very small proportion of mature individuals, i.e. *Chiloscyllium punctatum*, *Stegostoma fasciatum*, *Hemigaleus australiensis*, *Eusphyra blochii* and *Pateobatis hortlei* (Figs 2, 3 and 5). A few species were represented by a higher proportion of mature than immature individuals, i.e. *Carcharhinus fitzroyensis*, *C. macloti*, *R. taylori* and *Neotrygon annotata* (Figs 2, 3 and 5). Only a small number of species were represented by all size classes in the bycatch of the trawl fishery, i.e. *Carcharhinus coatesi*, *Gymnura australis* and *Maculabatis astra*.

### Sex ratios

For most species, sex ratios did not differ significantly from parity (χ^2^ test, *P* > 0.05). However, significantly more females than males were recorded for *Carcharhinus fitzroyensis* (3.2:1, *P* < 0.05), *Rhizoprionodon taylori* (1.9:1, *P* < 0.001), *Eusphyra blochii* (1.9:1, *P* < 0.01), and *Maculabatis astra* (1.5:1, *P* < 0.05). In contrast, significantly more males than females were recorded for *Carcharhinus coatesi* (2.2:1, *P* < 0.001), *C. limbatus* (4.5:1, *P* < 0.05), *Rhizoprionodon acutus* (2.1:1, *P* < 0.001), and *Pateobatis hortlei* (3.8:1, *P* < 0.005).

### Size at maturity

Size at maturity was calculated for those species with an adequate spread of data across the size classes for one or both sexes, i.e. *Hemigaleus australiensis*, *Rhizoprionodon acutus*, *Gymnura australis, Maculabatis astra* and *Aetomylaeus caeruleofasciatus*. Note that for the two most abundant shark species, the Australian blackspot shark *C. coatesi* and Australian sharpnose shark *R. taylori*, size-at-maturity of GoP populations will be provided in two manuscripts currently in review (L. Baje, unpublished data).

**Figure 6:**
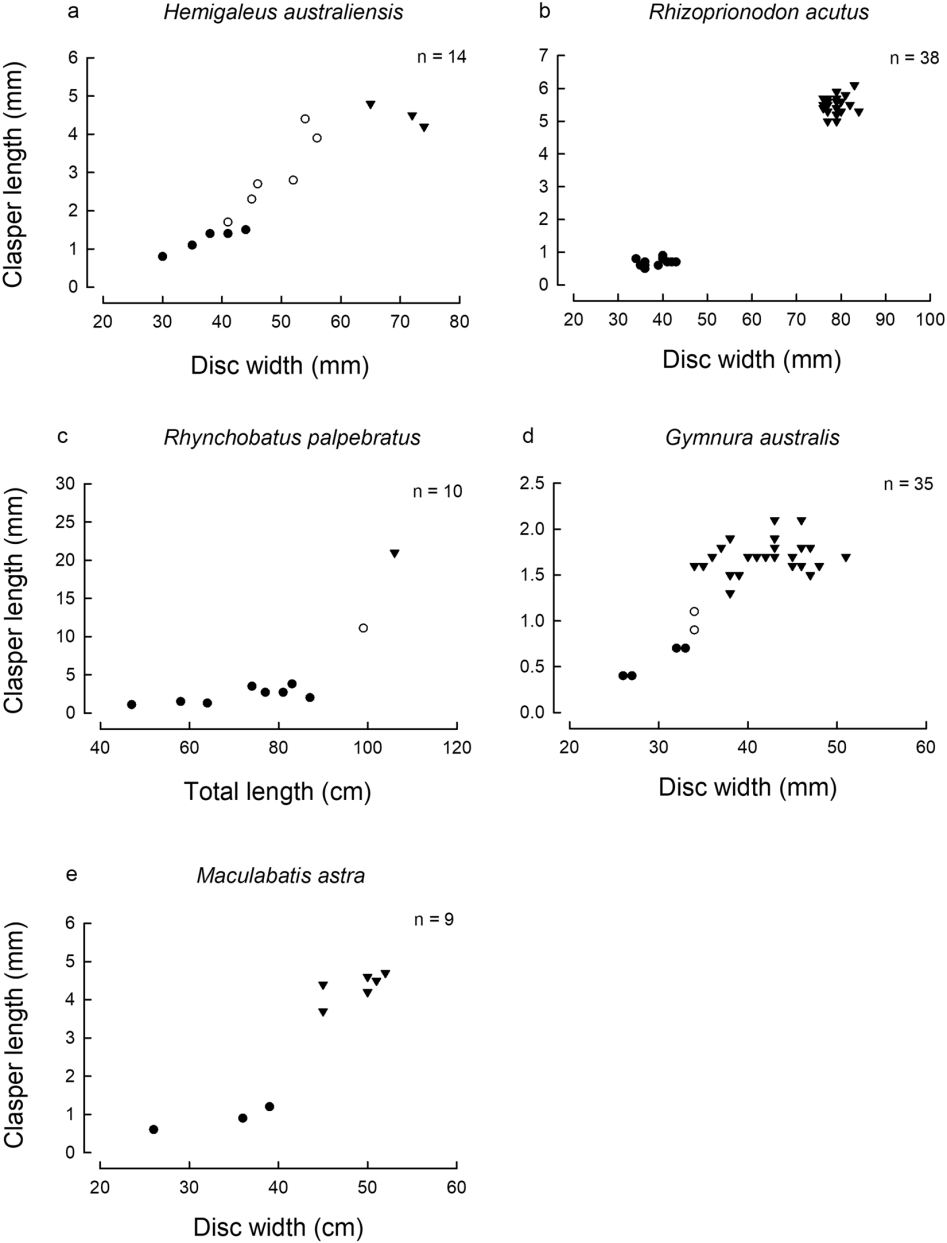
Clasper length vs. size (total length, TL or disc width DW) relationships for five species of sharks and rays: (**a**) *Hemigaleus australiensis*; (**b**) *Rhizoprionodon acutus*; (**c**) *Rhynchobatus palpebratus*; (**d**) *Gymnura australis*; and (**e**) *Maculabatis astra*.

Males of *Hemigaleus australiensis* below 56 cm TL possessed non- or partially calcified claspers, while claspers for specimens above 65 cm TL were all fully calcified (Fig. 6a). The *L*_50_ for males was calculated as 60.6 cm TL, but the high upper confidence value of 189.5 suggests the data were not adequate for a robust calculation. Claspers of *Rhizoprionodon acutus* below 43 cm TL were non-calcified, while all those above 76 cm TL were fully calcified (Fig. 6b). The *L*_50_ for males was calculated as 58.8 cm TL (57.0–62.2 cm TL). Only a single adult (106 cm TL) and a single subadult (99 cm TL) male *Rhynchobatus palpebratus* were recorded (Fig. 6c). Although an accurate *L*_50_ could not be calculated, size at maturity likely occurs between 99 and 106 cm TL.

All male *Gymnura australis* below 34 cm DW possessed non- or partially calcified claspers, while all specimens above 38 cm DW had fully calcified claspers (Fig. 6d). The *DW*_50_ for males was calculated as 34.2 cm DW (33.8–35.1 cm TL). All female *G. australis* below 48 cm DW were immature while all those above 52 cm DW were mature. The *DW*_50_ for females was calculated as 50.0 cm DW (46.2–52.1 cm TL).

All male *Maculabatis astra* below 39 cm DW possessed non-calcified claspers, while all those above 45 cm DW possessed fully calcified claspers (Fig. 6e). The *DW*_50_ for males was calculated as 41.5 cm DW (38.1–42.0 cm TL). Two male *Aetomylaeus caeruleofasciatus* of 34 and 37 cm DW possessed non-calcified claspers while two males of 45 and 48 cm DW possessed fully calcified claspers. The *DW*_50_ for males was calculated as 36.3 cm DW (30.0–40.2 cm TL).

### Spatial patterns in elasmobranch abundance

**Figure 7:**
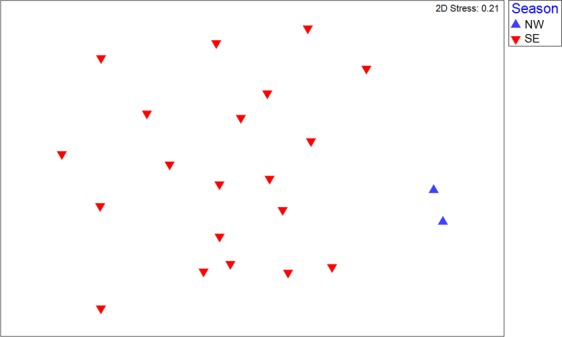
Non-metric multidimensional scaling (MDS) ordination of the elasmobranch catches in each of the fisheries management zones of the Gulf of Papua. Within each zone, each sample represents 5 randomly pooled trawl sets.

Catch composition between fishing zones was significantly different overall (ANOSIM, *P* < 0.001; *R* = 0.378; Fig. 7), and in most of the pairwise comparisons except zone 0 vs. zones 1 and 5, and zone 1 vs. all other areas (due to low sample sizes). The species most diagnostically different across zones were *C. coatesi*, *R. acutus*, *R. taylori*, *H. australiensis*, *M. astra* and *G. australis*, and to a lesser extent *E. blochii* and *C. brevipinna* (Table 4).

**Table 4:** Species identified by similarity percentages (SIMPER) as typifying fishing zones (bold text), and those species distinguishing each pair of fishing zones (normal text). Note, the low number of samples for zone 1 did not allow determination of typifying species and all pairwise comparisons were not significant based on ANOSIM results. Thus, zone 1 not included in this table. Also excluded from this table is zone 3 which did not have adequate trawls to include in analyses, and zone 4 where no elasmobranchs were caught in the three trawls undertaken.

Fishing zone	0	2	5	6	7	8
**0**	***C. coatesi***					
**2**	*C. coatesi R. taylori*	***R. taylori G. australis***				
**5**	*C. coatesi R. taylori*	*R. taylori C. coatesi*	***C. coatesi***			
**6**	*R. taylori C. coatesi*	*R. taylori E. blochii*	*R. taylori*	***R. taylori***		
**7**	*C. coatesi*	*R. taylori C. brevipinna*	*C. coatesi H. australiensis*	*R. taylori*	***H. australiensis G. australis R. acutus***	
**8**	*C. coatesi R. acutus*	*E. blochii R. taylori R. acutus G. australis*	*C. coatesi R. acutus G. australis*	*R. taylori R. acutus*	*R. acutus*	***G. australis M. astra R. acutus***

When comparing the number of each species caught per trawl in each of the fishing zones, clear species distribution patterns were apparent. *Neotrygon annotata* (n = 35) was only caught in the western half of the GoP (fishing zones 0–4), and not in the eastern half (fishing zones 5–8). Other species were far more abundant in the western than eastern half of the GoP: *E. blochii* (1.70 vs. 0.55 individuals/trawl), *Carcharhinus fitzroyensis* (0.31 vs. 0.06 individuals/trawl), *Rhynchobatus palpebratus* (0.81 vs. 0.46 individuals/trawl), *Hemitrygon longicauda* (0.72 vs. 0.19 individuals/trawl), *Himantura australis* (0.34 vs. 0.08 individuals/trawl), and *Pateobatis hortlei* (1.04 vs. 0.15 individuals/trawl).

In contrast, some species were only caught in the eastern half of the GoP (restricted to species with 5 or more individuals): *Stegostoma fasciatum* (n = 10), *Carcharhinus brevipinna* (n = 20), *Glaucostegus typus* (n = 5) and *Aetobatus ocellatus* (n = 5). The following species were also more abundant in the eastern than the western half of the GoP: *Chiloscyllium punctatum* (0.81 vs. 0.16 individuals/trawl), *H. australiensis* (0.97 vs. 0.27 individuals/trawl), *R. acutus* (1.88 vs. 1.14 individuals/trawl), *R. taylori* (3.63 vs. 2.88 individuals/trawl), *S. lewini* (1.42 vs. 0.33 individuals/trawl), *M. astra* (1.87 vs. 0.87 individuals/trawl), *Aetomylaeus caeruleofasciatus* (0.53 vs. 0.03 individuals/trawl) and *R. neglecta* (0.37 vs. 0.01 individuals/trawl).

### Temporal patterns in elasmobranch abundance

Elasmobranch catches were compared for trawls in the two seasons (NW and SE Monsoon) influencing PNG. There was no significant difference in catches of most species (ANOSIM, *P* > 0.05), although this was likely the consequence of the low abundances recorded for most species. However, significantly more individuals of the following species were caught in the SE Monsoon trawls than the NW Monsoon trawls: *C. punctatum* (χ^2^ test, *P* < 0.005), *H. australiensis* (χ^2^ test, *P* < 0.005), *C. brevipinna* (χ^2^ test, *P* < 0.005), *R. taylori* (χ^2^ test, *P* < 0.005), *S. lewini* (χ^2^ test, *P* < 0.005), and *R. neglecta* (χ^2^ test, *P* < 0.05). In contrast, significantly more individuals of the following species were caught in the NW Monsoon trawls than the SE Monsoon trawls: *C. coatesi* (χ^2^ test, *P* < 0.005), *R. palpebratus* (χ^2^ test, *P* < 0.005), *N. annotata* (χ^2^ test, *P* < 0.005) and *P. hortlei* (χ^2^ test, *P* < 0.05).

**Figure 8:**
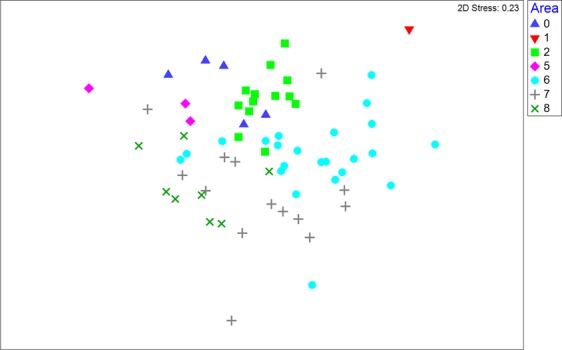
Non-metric multidimensional scaling (MDS) ordination of the elasmobranch catches in fisheries management zone 6 in both the Northwest (NW) and Southeast (SE) Monsoon seasons. Within each season, each sample represents 5 randomly pooled trawl sets.

Since the catch composition was shown to vary across fishing zones, particularly when comparing the eastern and western halves of the GoP, comparisons between the seasons were restricted to fishing zones with high numbers of trawls carried out in both seasons, i.e. zones 6, 7 and 8. Following ordination of the elasmobranch catches for fishing zone 6, the NW Monsoon samples (n = 2) formed a discrete group to the SE Monsoon samples (Fig. 8). ANOSIM demonstrated that the catch data across the two seasons were significantly different (*P* < 0.005; *R* = 0.464). SIMPER designated *R. taylori* to be the main species causing this difference, followed by *M. astra* and *C. coatesi*. It needs to be noted here that only 2 samples were available for the NW Monsoon samples. Additional samples from this zone during the NW Monsoon are needed to validate whether these differences are real. Similar ordinations for fishing zones 7 and 8 showed less discrete groupings of NW and SE Monsoon samples, and ANOSIM demonstrated that the catch data was not significantly different (*P* > 0.05) in both cases. Significantly more *R. taylori* were caught in the SE Monsoon period (0.57 individuals hr^−1^) across all zones than in the NW Monsoon period (0.24 individuals hr^−1^) (χ^2^ test, *P* < 0.005). More importantly, *R. taylori* was only recorded in the bycatch in zones 5–8 during the SE Monsoon, and not in any of the NW Monsoon trawls. It should be noted that although ~8 times more trawling occurred across these four zones in the SE Monsoon, it is still significant that no *R. taylori* were recorded in the 105 hours of trawling during the NW Monsoon in these zones. In contrast, 93 *R. taylori* were recorded in the trawl catches in zones 0–2 during the NW Monsoon (0.33 individuals hr^−1^ vs. 0.57 individuals hr^−1^ in zones 5–8 in the SE Monsoon).

When comparing day vs. night trawls, significantly more individuals of the following species were caught at night: *Chiloscyllium punctatum* (χ^2^ test, *P* < 0.005), *Hemigaleus australiensis* (χ^2^ test, *P* < 0.005), *Gymnura australis* (χ^2^ test, *P* < 0.05), *Hemitrygon longicauda* (χ^2^ test, *P* < 0.005) and *Rhinoptera neglecta* (χ^2^ test, *P* < 0.05). No species were caught in significantly higher numbers during the day. Ordination of the overall elasmobranch catches for fishing zones 2, 6, 7 and 8 showed mostly high overlap between day and night samples. ANOSIM demonstrated that the catch data were not significantly different (*P* > 0.05) between day and night in fishing zones 6, 7 and 8, but was weakly significant in fishing zone 2 (*P* < 0.05; *R* = 0.266). SIMPER designated *R. taylori*, *C. punctatum* and *R. palpebratus* to be the species most responsible for causing the difference between day and night trawls in fishing zone 2.

## Discussion

The large number of elasmobranch species recorded in this study, i.e. 18 sharks and 22 rays, reflects the high diversity of sharks and rays in PNG. Although the GoP prawn fishery only covers a relatively small proportion of PNG’s marine area, ~31% of the confirmed 130 elasmobranch species recorded from PNG^13^ were caught in this fishery. It should be noted that some larger and/or faster swimming species may not be captured in prawn trawl nets (see e.g.^14^). Since no Bycatch Reduction Devices (BRDs) or Turtle Excluder Devices (TEDs) are used in the GoP prawn fishery and the nets have stretched mesh size of ~50 mm, all bycatch encountered is likely to be retained in the trawl nets. PNG NFA is currently investigating the feasibility of introducing BRDs and/or TEDs into the GoP prawn fishery.

The closest comparable prawn trawl fisheries to the GoP prawn fishery is the Northern Prawn Fishery (NPF) in northern Australia and the Torres Strait Prawn Fishery (TSPF). The NPF operates over two fishing seasons, the first 2.5 months and second up to 4 months duration while the TSPF operates at night from 1 February to 1 December (http://www.afma.gov.au/fisheries/northern-prawn-fishery/). TEDs and BRDs were introduced into these fisheries in 2000^15^. In a study carried out before introduction of TEDs and BRDs^16^, the fish bycatch from 401 trawls was determined. The total trawling hours of this study was ~204 hrs vs. 1,273 hrs in the present study. They recorded a total of ~1,160 kg of elasmobranchs from 25 species and 11 families (vs. 4,368 kg from 40 species and 14 families in the present study), with a higher elasmobranch CPUE by weight than in the present study, i.e. 5.7 vs. 3.4 kg elasmobranchs hr^−1^. In a larger survey of the NPF, a bycatch of 56 elasmobranch species from 16 families was recorded^15^. A total of 35 of the 40 species recorded in the present study have also been recorded from the bycatch of the NPF^15^, highlighting the similarity in elasmobranch assemblages between the two regions.

The high elasmobranch species diversity in the GoP prawn fishery presents a challenge to monitoring and management of this fishery. The prawn trawl management plan^12^ states that “removing of fin from shark and returning to the sea alive is prohibited”, with no other policies regarding sharks and rays caught as bycatch. There was no evidence from the observer data that there were any breaches in this policy, however compliance levels when observers are not present are unknown. It is important to recognise the high level of mortality of sharks and rays in this fishery (81% of sharks and rays were dead at capture), due largely to the long haul times (up to 4 hrs) and lack of BRDs and TEDs. While TEDs are likely to exclude larger sharks and rays, particularly wedgefishes, the majority of the catches are small rays and sharks which are unlikely to be excluded^15^. Furthermore, the most threatened species, the sawfishes, are likely to still get entangled in the net or the TED itself. Thus, although the introduction of TEDs and BRDs into the GoP prawn fishery in the future could see a reduction in the capture of some sharks and rays, it is unlikely to reduce the majority of sharks and rays from being caught.

The susceptibility of the various shark and rays species caught in the NPF and TSPF in northern Australia has been investigated^15^. The species deemed most at risk and least sustainable in the NPF were the four species of sawfish (Pristidae) and *Pateobatis jenkinsii* (not recorded in GoP prawn fishery bycatch). A similar sustainability study and ecological risk assessment needs to be conducted on the elasmobranch bycatch in the GoP prawn fishery. It is likely that many of the larger species of sharks and rays are highly susceptible to trawling activities. However, it should be noted that a large portion of the Gulf of Papua is considered not suitable for trawling^11^, so there are likely to be refugia away from trawling activities for a number of species. Any assessment of the sustainability of elasmobranch species in the GoP prawn fishery needs to be extended to incorporate the impact of other fisheries operating in the GoP. In particular, various coastal fisheries, e.g. gillnetting and seine netting, capture many of the same species caught as bycatch in the GoP prawn fishery (W. White, unpublished data). The limited information available for these fisheries indicates that many species overlap with the GoP prawn fishery.

The significantly lower CPUE in the eastern half of the GoP may be due to a number of factors. Firstly, the western half of the GoP is adjacent to enormous river outflows, e.g. Fly, Kikori and Purari rivers, with presumably higher productivity compared to the eastern half of the GoP. Thus, the western half may naturally have higher abundances of elasmobranchs, but there are no data that bear on this question. An alternative explanation is that the much higher trawling pressure in the eastern half of the GoP, e.g. 70% of trawls recorded in this study were in sites 6–8, has resulted in lower abundances of elasmobranchs through overexploitation. However, without a historical time series of elasmobranch CPUE data from the GoP prawn fishery, it is not possible to determine the impacts of trawling on elasmobranch populations and species in the GoP.

The trawl data have contributed to knowledge of elasmobranch diversity in the region. One species of shark (*C. fitzroyensis*) and two species of rays (*M. microps* and *U. acanthobothrium*) recorded during this study were the first records of these elasmobranch species in PNG waters^13^. Furthermore, specimens of *H. australis* and *A. caeruleofasciatus* retained from the trawl catches contributed to the description of these two recently described species, both also found in northern Australia^17,18^.

The presence of only immature individuals of *C. brevipinna*, *C. limbatus* and *S. lewini* in the trawl bycatch suggests that these species may have nursery areas within the GoP, but this suggestion needs to be evaluated rigorously based on criteria that are used to positively identify nursery locations^19,20^. These three species have been recorded as using inshore nursery areas for their young elsewhere in their wide ranges, e.g. off South Carolina in the USA^21–23^. Only immature individuals of *H. longicauda* were also recorded. New-born and juvenile individuals of this species are regularly caught in the intertidal zone by seine net fishers along the Western and Gulf Provinces (W. White, unpublished data) and adults are largely unknown. Recently, several images of adult specimens have been taken from shallow waters in the Western Province of PNG (J. Page, pers. comm.). Adults of this species may occupy different, possibly more specific habitats than the juveniles outside of the regularly trawled area in the GoP.

The full size cohort of a number of elasmobranchs were recorded in the trawl bycatch in the GoP indicating that they not only give birth in this area, but occur in these inshore waters at all stages of their life cycle. The most abundant sharks which fall into this category were *C. punctatum*, *H. australiensis*, *C. coatesi*, *R. acutus*, *R. taylori* and *E. blochii* which were represented in the trawl bycatch by a wide size range of individuals from juveniles to adults. Similar distributions are known for these species from northern Australia where they also occur^24,25^ (in^25^ two species under the older names *H. microstoma* [=*australiensis*] and *C. dussumieri* [=*C. coatesi*]). The most abundant rays represented in the catch by a wide size range were *R. palpebratus*, *G. australis*, *M. astra* and *A. caeruleofasciatus*. All size ranges of these four ray species were also represented in the trawl bycatch in Australia’s northern prawn fishery^15^ (in^15^ three species under the older names *R. djiddensis* [presumably mostly *R. palpebratus*], *Himantura toshi* [=*M. astra*], and *A. nichofii* [=*A. caeruleofasciatus*]).

Although a number of species were caught in significantly different numbers in the two seasons examined, the SE Monsoon and NW Monsoon, the effects of fishing zone also needed to be taken into account. The most informative seasonal information came from comparing catches in the two seasons from within a fishery management zone. Results showed that the biggest difference between seasons was the lack of catches of *R. taylori* in the eastern zones during the NW Monsoon. Off northeastern Australia, *R. taylori* was found to prefer seagrass habitats but between December and February they abruptly move to sandy inshore habitats coinciding with increased river discharge^26^. This may account for the seasonal differences in *R. taylori* catches in the GoP. In the NW Monsoon, increasing river discharges into the GoP may have a similar effect, causing *R. taylori* to select more inshore, sandy habitats where trawling is limited. As more river outflows are present in the western half of the GoP, this would account for catches of *R. taylori* in zones 0–2 during the NW Monsoon but not in zones 5–8. The western half is also shallower so is likely a combined effect of *R. taylori* populations moving more inshore and westward during the NW Monsoon. Unfortunately no trawls in zones 0–2 were recorded in this study during the SE Monsoon to determine whether the catches remained stable or differed significantly according to the season. More detailed seasonal catch data is required from within each of the management zones to confirm the above hypotheses regarding *R. taylori* and to determine other seasonal patterns.

Although only weakly significant or no differences were seen in catches between day and night-time trawls, several species were caught in significantly higher numbers at night than during the day, i.e. *C. punctatum, H. australiensis, G. australis, H. longicauda* and *R. neglecta*. In contrast, no species were caught in significantly higher numbers during the day. It is possible that these species undergo diel movements between shallower, inshore waters and the deeper coastal waters where trawling is more prevalent. There is limited data on diel patterns in these species from trawl bycatch data elsewhere or more generally in similar habitats. More detailed bycatch data from the GoP, ideally coupled with comparative inshore catch composition data, are needed to be able to investigate diel patterns in more detail.

The GoP prawn fishery bycatch includes a wide diversity of shark and ray species ranging in size from only 12 cm in width to over 3 m in length. Prior to this study, no data existed on the elasmobranch bycatch of the GoP prawn fishery in PNG. This study provides crucial baseline data for PNG for the fishery. The introduction of BRDs and TEDs in the future will likely benefit at least the larger size classes of a number of species that are currently landed in this fishery^15,27^. The long average duration of the trawl hauls in the GoP prawn fishery contributes to the high catch mortality of sharks and rays which needs to be considered by fisheries managers. The removal of fins from most sharks and use of the meat of some species possibly makes them a desirable bycatch which should also be considered by fisheries managers. Further research and monitoring of the GoP prawn fishery shark and ray bycatch is required coupled with more comprehensive data from the coastal fisheries adjacent to the fisheries management zones.

## Methods

### Study area

The GoP is located on the southern coastline of mainland PNG and is adjacent to the northwestern margin of the Coral Sea. It has a total area of ~30,000 km^2^ and consists of a broad shelf with a maximum width of ~150 km near the Fly Delta and narrows to less than 20 km east of the Purari River delta^28^. The southwestern tip of the GoP joins the shallow Torres Strait shelf of northeastern Australia. The numerous rivers which flow into the GoP, including the massive Fly River system, discharge ~1.5 billion tonnes of sediments each year^28^. Within the GoP, muddy to sandy bottoms are prevalent to about 50 m depth, creating a large area suitable for trawling. Between 50 and 70 m, large rocky peaks occur which are unsuitable for trawling and beyond about 80 m depth, the sea floor rapidly drops off, making trawling difficult^11^.

The Gulf of Papua prawn fishery management zone is divided into 8 zones in the coastal zone of the GoP: 1 – North Fly; 2 – Cape Blackwood; 3 – Purari; 4 – Orokolo Bay; 5 – West Kerema Bay; 6 – Kerema Bay; 7 – Freshwater Bay; 8 – Iokea^29^ (Fig. 1). Some trawling also occurs off the Fly River mouth southwest of area 1; this area is referred to as 0 – Fly. The trawl zones from Cape Blackwood to Iokea (2–8) are closed from fishing between 1^st^ December and 31^st^ March each year under the current Gulf of Papua Prawn Fishery Management Plan^12^.

### Sample collection

This work is a collaboration with the National Fisheries Authority (NFA), the government agency responsible for managing commercial fisheries and implementing fisheries research in PNG. Fishery observers were deployed on-board prawn trawlers with the task of identifying and recording sharks and rays that were observed in the bycatch as well as additional parameters described below. The sharks recorded and/or collected by observers in this study had already suffered mortality in the process of fishing and subsequent landing, and no sharks were intentionally sacrificed for the study. Although some sharks and rays were alive when the nets were emptied on the vessel deck, they had all suffered mortality before observers were able to handle them. All sampling procedures were allowed by the NFA. No further permits were required by relevant authorities.

All data were obtained by PNG NFA observers during commercial prawn trawling activities. The PNG NFA observer program consists of well-trained observers (both by the Secretariat of the Pacific Community fisheries program and the current project) with detailed data collection protocols and species identification guides. Five PNG NFA observers were deployed on 7 commercial prawn trawl trips (across 5 vessels) between June 2014 and August 2015. Details of the 5 trawl vessels on which observers were placed are provided in Table 5. Twin- or quad-rigged trawls were deployed, with the total sweep of both of these rigging types being 60 m^8^. A small ‘try’ net was deployed during the trawls and was checked every 15 minutes. When the try net indicated suitable prawn catches, the vessel trawled along that depth contour for up to 4 hours^8^. The mesh size used in the nets is ~50 mm stretched mesh with no mesh size less than this size allowed to be used in this fishery to catch prawns^12^. During a fishing trip, trawling occurred on a 24-hour basis and each vessel trawled for about 250 days per year during the open season^8^. Since only a single observer was deployed per fishing trip, observer coverage was restricted to around 12 hours per day, i.e. about 4 trawls per day. Bycatch data used in this paper is available from the Dryad Digital Repository: 10.5061/dryad.f77972k. All data used in this study were collected by PNG National Fisheries Authority personnel who are the approved national authority for fisheries research in PNG.

**Table 5:** Specifications of the five trawl vessels on which observers were deployed on in this study (from^8^).

Vessel	Length (m)	Gross Registered Tonnage	Main Engine (kW or HP)	Rigging
FV Ipali	21.36	138.07	388 kW	Quad
Charisma	21.36	138.07	388 kW	Quad
Lavai No. 1	27.83	150.07	420 kW	Twin
Lou Aro	27.83	150.07	420 kW	Twin
FV Siwi	29.3	113.67	540 HP	Quad

### Elasmobranch species identification

All sharks and shark-like rays less than ~80 cm total length (TL) and rays less than ~40 cm disc width (DW) were retained whole and frozen for subsequent processing in Port Moresby. All larger specimens were photographed, measured and sexed on the deck and a genetic sample was taken and stored in ethanol for subsequent DNA analysis if required. Sampling processes enabled the identification and accurate verification of all sharks and rays caught during trawling. For specimens that could not be accurately identified from the images taken by the observers, genetic techniques were employed to confirm identifications. To allow for the best comparisons with other species, the NADH2 mitochondrial marker was sequenced and compared with other specimens in the Chondrichthyan Tree of Life database (https://sharksrays.org). Amplification and sequencing of the NADH2 gene followed the protocols outlined in^30^.

### Catch composition

For each specimen, the horizontal stretched total length (TL; for all sharks and shark-like rays, i.e. guitarfishes, wedgefishes and sawfishes) or disc width (DW; for all stingrays, eagle, butterfly and cownose rays) was recorded. Sex was also recorded and for males, the degree of calcification of the claspers and outer length of the claspers was recorded where possible. However, clasper length was only recorded for a subset of those males for which clasper calcification was recorded. For specimens frozen on-board, processing was undertaken at a laboratory at the Biological Sciences building at the University of Papua New Guinea. As well as TL or DW, total weight, female maturity (where possible), and clasper outer length was also recorded. Internal male maturity stage, e.g. testes development and degree of coiling of vas deferens, were not recorded due to time constraints and the fact that claspers provide an accurate means of determining maturity in males. The number, sex and size of embryos was recorded from pregnant females. Significance of sex ratios for species with more than 10 sexed individuals across the fishing zones were tested with χ^2^ test in Microsoft^TM^ Excel. The condition (dead, dying or moribund, or alive) at time of landing on the vessel deck of 924 of the 2,030 elasmobranchs caught was recorded by observers.

### Maturity analyses

For species with adequate maturity data (>15 individuals with maturity recorded representing both juveniles and adults), the size (TL or DW) at maturity of females and males was calculated. For males, individuals with either non-calcified or partially calcified claspers were considered immature, while those with fully calcified claspers were considered mature^31^. For females, individuals without mature uteri and ovaries were considered immature and those with mature ovaries and uteri were considered mature^31^. The size, at which 50% of females or males of a particular species attain maturity (Size_50_) was derived using logistic regression, where the proportion, *P*, of those sharks that were mature at size *S* was calculated as,

$$P=\frac{1}{1+\exp [-\,\mathrm{ln}(19)\frac{(S-{S}_{50})}{({S}_{95}-{S}_{50})}]},$$

where S_50_ and S_95_ are the sizes at which 50 and 95% of the individuals, respectively, were mature. Maximum likelihood estimates of the parameters were obtained using the routine SOLVER in Microsoft^TM^ Excel. Reported estimates of parameters were determined as the median values derived from 200 sets of randomly resampled data, with the same sample size, drawn from the data on the observed maturity status at size for individuals. The approximate 95% CI were estimated as the 2.5 and 97.5 percentiles of the 200 estimates resulting from these resampled data^32^.

Clasper (outer) length vs. body size relationships were produced for species with adequate samples sizes of immature and mature individuals. Note that only a subsample of specimens which had clasper calcification recorded also had clasper length measured.

### Calculating biomass

The weight of each individual measured but not weighed was calculated using a power curve (*W* = *aL*^*b*^) derived from individuals that were measured and weighed. For those species without adequate numbers of measured and weighed individuals, *a* and *b* parameters were obtained from published sources. If no published parameters were available, those from a morphologically similar species were used to estimate biomass, including: *Neotrygon annotata* parameters used for *N. picta*, *Aetobatus narinari* parameters used for *A. ocellatus* and *Mobula alfredi*, *Himantura australis/leoparda* parameters used for *Pateobatis fai*, *Pastinachus ater*, *Urogymnus acanthobothrium*, *U. granulatus* and unknown stingray. The weight of the single *Megatrygon microps* recorded (~180 cm DW) was conservatively estimated to be ~80 kg based on a published weight of 75 kg for a 170 cm DW specimen from Iranian waters^33^.

### Spatial and temporal multivariate analyses

The number of each elasmobranch species in each trawl haul in each of the fishing zones (0–8) was determined. Single trawl hauls often only contained a few of the total elasmobranch species recorded, which was problematic for multivariate analyses. To overcome this, data were pooled for groups of trawls, similar to what is done with stomach content data^34,35^. Thus, the catch data were randomly allocated into groups of five trawl hauls within each of the fishing zones and mean catch composition values determined. These pooled samples were subjected to non-metric multidimensional scaling (MDS) ordination in order to determine whether fishing zone influenced the elasmobranchs species composition. Prior to subjecting the pooled catch data to MDS ordination, data were square-root transformed and a similarity matrix was constructed using the Bray-Curtis similarity coefficient and ordination in PRIMER v7 package following the techniques outlined in^36^. One-way analyses of similarities (ANOSIM) were used to test whether the elasmobranch catches differed significantly amongst fishing zones. Similarity percentages (SIMPER) were employed to determine the elasmobranch species that typified particular fishing zones and/or contributed most to the dissimilarities between zones^37^. To remove the effect of abundances of the various species to the ordination, a separate analysis with a presence/absence transformation was also undertaken.

To determine the effect of season (Northwest Monsoon, NW, between November and April; or Southeast Monsoon, SE, between May to October) on catch composition, means of randomly allocated groups of five trawl hauls within either season within each fishing zone were produced and analysed as described above. Similarly, to determine the effect of day vs. night on catch composition, means of randomly allocated groups of five trawl hauls within day or night within each fishing zone were produced and analysed as above.

To test for differences in the catch per unit of effort (CPUE, number per hour trawled) of elasmobranchs a generalised linear mixed model (GLMM) with Fisheries Management Zone, Season (NW monsoon/SE monsoon) and Time of Day (day/night) as factors was used. Zones 3 and 4 had limited data and were thus excluded from the analyses. To account for differences in fishing power and observer ability between vessels Fishing Trip was included as a random factor in the model. No interactions between factors were tested due to limitations in the data set. CPUE values were square root transformed to meet assumptions of normality in the data.
